# Effect of Graphene Nano-Additives on the Local Mechanical Behavior of Derived Polymer Nanocomposites

**DOI:** 10.3390/polym10060667

**Published:** 2018-06-15

**Authors:** Mostapha Tarfaoui, Khalid Lafdi, Imane Beloufa, Debora Daloia, Ali Muhsan

**Affiliations:** 1 ENSTA Bretagne, IRDL—UMR CNRS 6027, F-29200 Brest, France; imane.beloufa@ensta-bretagne.org; 2 University of Dayton, Dayton, OH 45469-0168, USA; klafdi1@udayton.edu (K.L.); ddaloia@gmail.com (D.D.); muhsana1@udayton.edu (A.M.)

**Keywords:** epoxy matrix, graphene nano-additives, micro-indentation test, local properties, effect of graphene

## Abstract

In this study, indentation tests of graphene-based polymer nanocomposites were carried out to determine the local elastic mechanical properties. The samples consist of epoxy matrix with graphene additives. Additives were added at levels of 0% as a control, 0.5%, 1%, 2.5%, 5% and 10% by weight. The local elastic properties such as moduli and hardness were calculated. After each indentation, the prints were characterized using scanning electron microscopy (SEM). It seems that the local mechanical properties of nanocomposite samples were improved as the amount of nano-additives increased. Based on the curve displacement and surface imaging, we can conclude that the nano-additives influenced the overall plastic mechanical behavior of the samples. For simulating micro-indentation test, a finite element analysis model was developed using ABAQUS software and compared to experimental tests. Good correlation was observed.

## 1. Introduction

To enhance the mechanical performance of monolithic epoxy, many researchers have used nanofillers such as graphene, carbon black, carbone nanotubes, and other carbonaceous materials to reinforce it, due to their high modulus of elasticity and capacity to stop crack propagation [1,2,3]. Indeed, graphene has become popular as a reinforcement material for a wide range of polymer matrices, including polystyrene [4], epoxy [5,6,7], polyaniline [8], polymethylmethacrylate [9], polypropylene [10] and nylon [11], for several applications. It can improve the thermal stability, mechanical and electrical properties of polymers. Furthermore, they are affordable [12]. Atif et al. [13] published a review paper concerning the effect of graphene nanoparticles and their influence on thermal, electrical and mechanical properties. They showed that adding a small amount of nanoparticles seems to improve the overall physical properties. The incorporation of graphene in composites can increase its fracture toughness by as much as 131%. It was observed that the graphene distribution, its weight fraction and its surface modification all have a strong influence on the properties of nanocomposites. However, all these parameters contribute to the degree of dispersion of these additives in the polymer matrix. In recent studies, several processing routes were proposed to disperse graphene additives into a polymer, e.g., melt compounding, solution blending, and in situ polymerization [14]. Kim et al. [15] observed that these three different processing methods produced different effects on the mechanical properties and electrical conductivity of graphene/polyurethane composites, which might lead to different dispersion levels of graphene in the matrix. On the other hand, Tang et al. [16] used two different dispersions of graphene, with and without ball mill mixing, to investigate the influence of graphene exfoliation on the mechanical properties of epoxy resin. They found a negligible difference in both the tensile and flexural modulus. However, a poor dispersion of epoxy/graphene showed a lower strength and fracture toughness than a good dispersion. Furthermore, Yao and colleagues [17] studied the homogeneous dispersion of graphene nanosheets in epoxy to facilitate mixing process, and favor stability of graphene via chemical functionalization. On the other hand, they showed that graphene nanosheets are well-exfoliated in epoxy using optical and transmission electron microscopy. The influence of modified graphene on the tensile and thermal properties of epoxy/graphene is studied to comprehend their property–structure relationship. The enhancement in mechanical properties of nanocomposites is not only due to the dispersion but also to the type of graphene and the interfacial bonding between graphene and epoxy resin as confirmed by microscopy characterization [18]. Salom et al. studied the influence of various types of graphene on the adhesive and mechanical properties of an epoxy resin [19]. In their research, three different types of graphene were used to prepare epoxy/graphene-based nanocomposites: two non-functionalized graphene having different sizes and one functionalized using amine groups. All nanocomposites showed higher moduli than the neat epoxy. whereas increase of graphene presented lower lap shear strength due to the aggregation of the graphene particles. Alexopoulos et al. [20] investigated the mechanical performance of an epoxy resin reinforced with different concentrations and sizes of graphene nanoplatelets. To increase the interfacial interaction between the resin and nanofillers, an epoxy was prepared with hydroxyl functionalized graphene by Manjunatha et al. [21]. They showed that the epoxy resin contains 0.5 wt % of functionalized graphene present a higher strength compared with neat epoxy and specimens having 0.25 and 1.0 wt % of nanofillers. Ferreira et al. [22] studied the functionalized graphene oxide as reinforcement in epoxy based nanocomposites. The results showed improved properties in the modified surface with finding an improvement of hardness by 33% for functionalized graphene oxide.

Indentation has been intensively used to characterize local mechanical properties of different materials such as polymer, composites, etc. The aim of this study was to quantitatively understand the effects of neat epoxy and NGP/epoxy on its indentation measurement with experimental and numerical approaches. Micro-indentation tests of graphene-based polymer nanocomposites were carried out to determine the local elastic mechanical properties. The samples consisted of epoxy matrix with graphene additives. Additives were added at levels of 0% as a control, 0.5%, 1%, 2.5%, 5% and 10% by weight. Our results clearly demonstrate that the influence of the presence of NGP in epoxy matrix on its indentation measurement can be significant.

## 2. Materials and Sample Preparation

**Figure 1:**
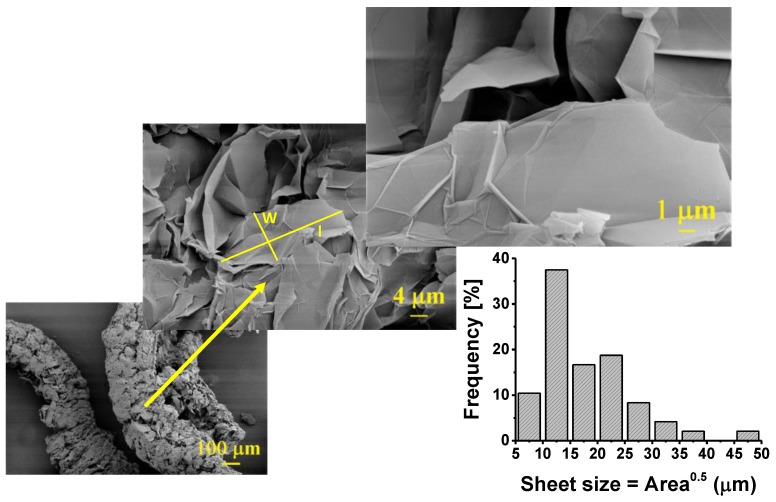
SEM image of the graphene additives and their size distribution.

**Figure 2:**
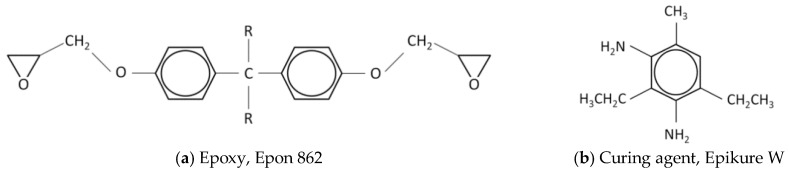
Molecular formulas.

The nanocomposite samples were produced using the conventional dry mixing method and adding 0.5, 1, 2.5, 5 and 10 wt % of graphene to epoxy. To maintain uniformity and produce sound samples, the unfilled polymer and the composites were processed under identical conditions. Graphene used in this study was produced by exfoliation and reduction of graphite in our own laboratory (Figure 1). The polymer was a low viscosity liquid epoxy resin, Epon 862 (Diglycidyl Ether of Bisphenol F), and the curing agent was Epikure W (diethyl toluenediamine, or DETDA), both acquired from Momentive Specialty Chemicals Inc. (Cleveland, OH, USA). This epoxy was chosen here due to its low viscosity and long shelf life at room temperature, which make the system versatile and easy to process. The aromatic amine curing agent has the advantage of providing excellent performance and chemical resistance at elevated temperatures. The molecular formulae of both compounds are given in Figure 2.

Graphene tends to agglomerate in chunks because of its small particle size, large surface area and superior particle activity. To improve the dispersion of graphene in epoxy resin, the graphene bundles were dry-blended in a blender to obtain a uniform density graphene. It was then weighed to obtain five concentrations ranging from 0 to 10 wt % with the epoxy resin system. The lowest graphene concentration selected was 0.5 wt %, an intermediate value of 5 wt %, and the maximum concentration used was 10 wt %. It was found that, for concentrations higher than 10 wt %, the mixing process became challenging and a well dispersed compound was very difficult to obtain.

**Figure 3:**
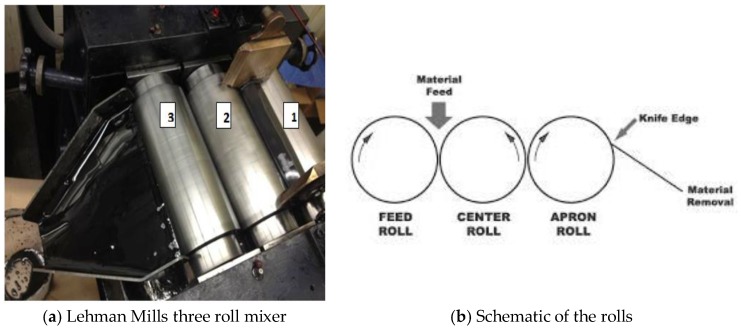
The mixture production device.

The nanofiller was added, based on the desired filler concentration, to the epoxy resin, which was previously mixed with the curing agent in a plastic container by hand for 5 min in a weight ratio of 100/26.4 (epoxy/curing agent). This blend was subsequently loaded in a three-roll mill mixer to obtain a homogeneous dispersion of graphene in the epoxy. The procedure was the same for all compositions, with the exception of 10 wt % of graphene. As the amount of nanofiller increased, the viscosity of the compound increased. Therefore, the 10 wt % compound was prepared in several steps. Here, a small amount of graphene was added to the resin, then it was mixed in the three-roll mill until the viscosity of the compound was reduced. Subsequently, more graphene was added. This step was repeated until all the graphene was incorporated in the resin. A three-roll mixing mill, from Lehmann Mills (Figure 3a), was employed to mix graphene in epoxy and obtain a uniform mixture. According to Gojny et al. [23], a laboratory scale three-roll mill is the most capable equipment to uniformly mix the nano-fillers in polymers. The three horizontal rolls rotate in opposite directions at different speeds relative to each other, by means of an electric motor and gear box assembly. The rotational speed of center and apron rolls (Figure 3b) is increased to apply large shear stress on the mix and improve mixing and dispersion. The standard speeds for rollers 1, 2, and 3 were 21, 64 and 200 rpm, respectively. The gap between the rolls was kept as small as possible to produce large amount of shear stresses. Here, a feeler gauge was used to set a 25 μm gap between the rolls. It was further decided that for all formulations, the compound would be passed through the roll mill five times, and the feed roll speed be increased with increasing graphene content (Table 1). Since the shear stress produces heat, the temperature of the rolls was controlled by a cooling system, where chilled water at a temperature of 21 °C was used.

**Figure 4:**
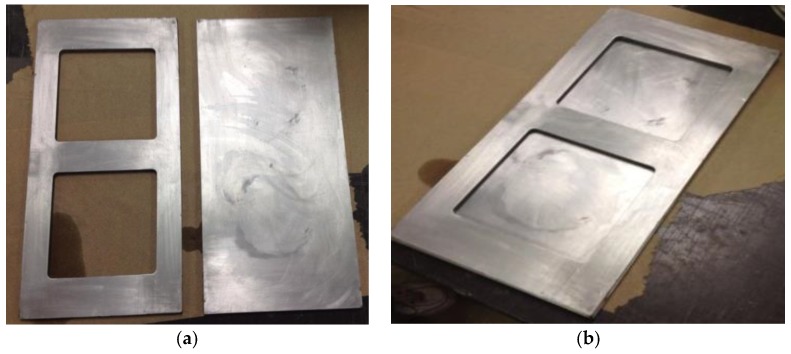
(**a**) Two-separate piece mold made of stainless steel; (**b**) Combined molds one serves as a window frame and the second as a baseplate.

The mixing of the compound was started by slowly pouring the pre-mixed compound (graphene, epoxy and curing agent) between feed and center rolls. The mixture then automatically passed between center and apron rolls by sticking to them, and a knife edge blade scraped the processed material off the apron roll and stored in the pan (Figure 3). When the entire mixture was collected in the pan, it was poured again between the feed and center rolls. The process was repeated five times for each compound. As the mixture became homogeneous, it started presenting a shiny metallic surface due to the presence of exfoliated graphite. Composite samples were then molded into 10 cm × 10 cm × 0.5 cm plates using a two-piece mold, as shown in Figure 4. The mold was comprised of two flat plates (aluminum) and a 5 mm thick aluminum plate, which had two 10 cm × 10 cm windows machined in it. First, the mold release agent, Frekote 770-NC from Henkel (Germantown, WI, USA), was applied on all three pieces of the mold, which were previously cleaned and dried. Two coats of this agent were applied and, after the evaporation of the solvent, the composite mixture was poured into the assembled mold (aluminum and molding plate) (Figure 4b), which were held together by high temperature tape. The poured mold was then covered with the silicone rubber plate and transferred to the molding press. This mold was heated to a temperature of 121 °C for 10 min without applying pressure to eliminate any bubbles, and then a load of 7 tons was applied on the mold. According to the technical data sheet, the curing process called for: heating the mold under pressure for 1 h at a temperature of 121 °C, 2 h at 177 °C and finally cooling down to 38 °C prior to releasing pressure. The cooling of the mold inside of the press was an effort to obtain a flat sample.

Afterwards, both sides of the composite plates were polished by hand for 1.5 min each with a 180 grit emery paper, and then they were washed in water and dried.

The mechanical properties of each constituent are listed in Table 2.

## 3. Indentation Tests

**Figure 5:**
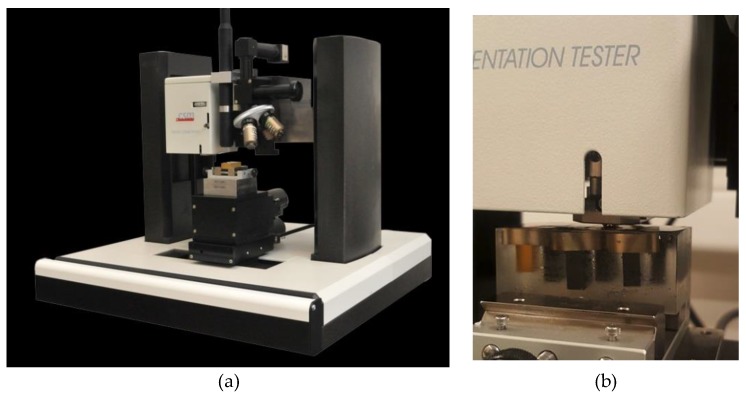
Image of (**a**) the indentation machine and (**b**) specimens under test.

The mechanical properties of epoxy matrix with grapheme additives specimens were evaluated for each volume fraction using the indentation test. A CSM Micro-Hardness Tester (Figure 5) using Vickers diamond indenter with a nominal angle of 136° was used in this study. The micro-indentation parameters used for the tests are: approach speed of 50,000 nm/min, contact load of 20 mN, load rate of 2000 mN/min, unload rate of 2000 mN/min, maximum load of 1000 mN and 20 s of pause. Schematic representations of indentation curves are presented and analyzed in this section for different graphene weight fractions.

**Figure 6:**
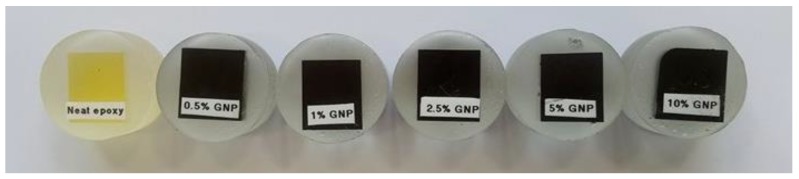
Micro-indentation tests.

For this type of material, and to control the dispersion of graphene in the matrix, micro-indentation tests were carried out in the specimen (Figure 6). Minimum distances of 0.5 mm between indentation marks were used to avoid any hardening effect from previous tests. Possible indentation forces are ranging from 1 mN to 10 N. The elastic moduli, stiffness, hardness, and max displacement were obtained as the average value of all specimen tests. For a rigorous approach and a realistic comparison, the testing conditions are the same for all specimens and for all weight fractions.

## 4. Results

### 4.1. Graphene Dispersion in Epoxy

**Figure 7:**
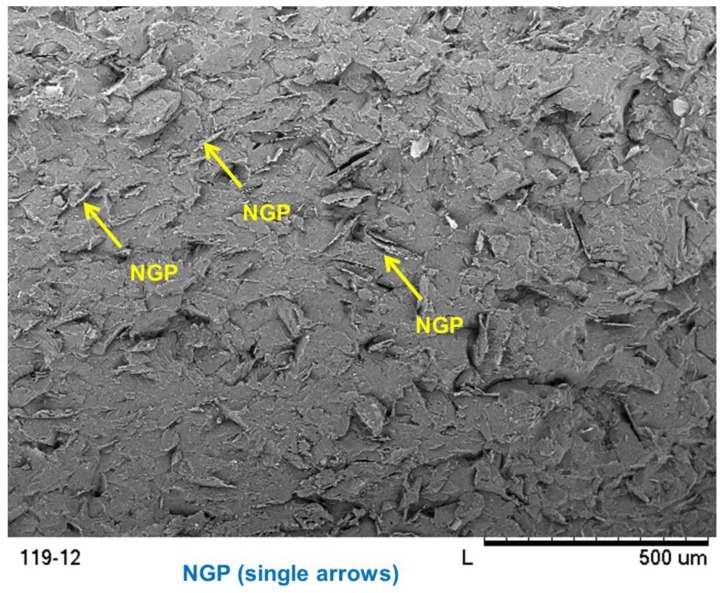
Overview nanocomposite fracture surfaces showing the graphene dispersion.

The mechanical and physical properties of nanocomposites depend on the dispersion of filler agent into the matrix. Here, the dispersion of graphene in epoxy was analyzed using optical and scanning electron microscopes. The optical microscope specimens were produced by mounting a 5 mm × 5 mm × 5 mm composite cube in epoxy holder and polishing its surface to optical flatness through several steps. The SEM samples were obtained by breaking a thin strip of the composite in tension. The graphene layers in epoxy can be identified via color contrast caused by the polarized light. This method is qualitative because the color contrast often varies from one laboratory to another [19]. However, it is an accurate and reliable technique to evaluate the graphene dispersion and distribution in polymer matrix. Figure 7 shows optical microscope images of cured nanocomposites for 1, 5 and 10 wt % of graphene filled epoxy. The white spots and streaks as indicated by arrows are the graphene sheets. In all cases, the graphene can be seen to be dispersed all over the surface. The density of graphene sheets increases with its concentration. As such, these images indicate that the graphene dispersion in epoxy is reasonably uniform, which was further verified by the SEM analysis.

**Figure 8:**
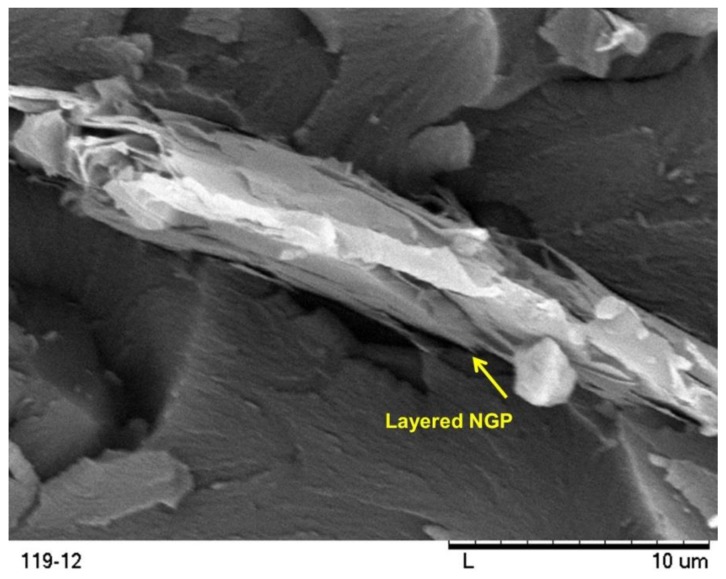
Enlarged view of nanocomposite fracture surfaces showing graphene morphology.

Figure 7 and Figure 8 show the scanning electron micrographs of the fracture surfaces of graphene filled epoxy strips. Figure 7 shows the overview of the nanocomposites identifying the graphene sheets, while Figure 8 provides the detailed view of each of the surfaces. In both cases, the nanographene sheets (NGP) are again indicated by single arrows. It may be noted that graphene is reasonably well dispersed in all the three composites. The wrinkled surface morphology of the graphene nanosheets is shown in Figure 8. According to Shen et al. [24], it is this morphology which plays an important role in enhancing mechanical interlocking and transferring load from the epoxy matrix to graphene sheets.

### 4.2. Graphene Nanoparticles (NGP) Effect

**Figure 9:**
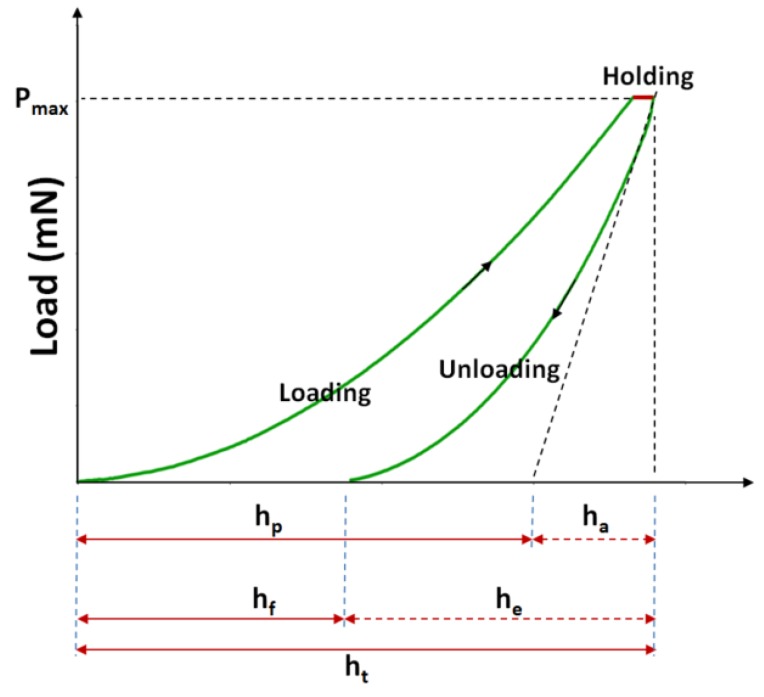
Representative plot of load versus displacement.

The mechanical properties of epoxy matrix with graphene additives specimens were evaluated for each weight fraction using an indentation test. In this study, a CSM Micro-Hardness Tester with a Vickers diamond indenter was used. A minimum of eight indentation tests were carried out for every sample. Minimum distances of 0.5 mm between indentation marks were used to avoid any hardening effect from previous tests. For all the micro-indentation tests carried out, the maximum effort was set 1000 mN. The micro-indentation parameters used for the tests are: approach speed of 50 μm/min, contact load of 10 mN, load rate of 2000 mN/min, unload rate of 2000 mN/min, maximum load of 1000 mN and 20 s of pause. The elastic moduli, indentation depth, stiffness and hardness were determined as an average value of all specimen tests. For a rigorous approach and a realistic comparison, the testing conditions were the same for all specimens. Micro-indentation tests were performed using Vickers indenter, which monitors and records the load vs. displacement of the indenter. Figure 9 shows the four steps of a micro-indentation tests:

- First step: Vickers indenter approaches the surface.
- Second step: Loading phase up to the maximum load. The maximum load applied was 1000 mN.
- Third step: Holding the load. It was done to avoid the creep effect on the unloading characteristics.
- Fourth step: Unloading phase.

The indentation depths:

h_t_ is the total depth under a load, Pt;
h_e_ is the elastic rebound depth during unloading;
h_f_ is the residual impression depth;
h_a_ is the surface displacement at the perimeter; and
h_p_ is the contact indentation depth.

The contact stiffness, *S*, is defined as the slope at the beginning of the unloading curve.

### 4.3. Micro-Indentation Tests

**Figure 10:**
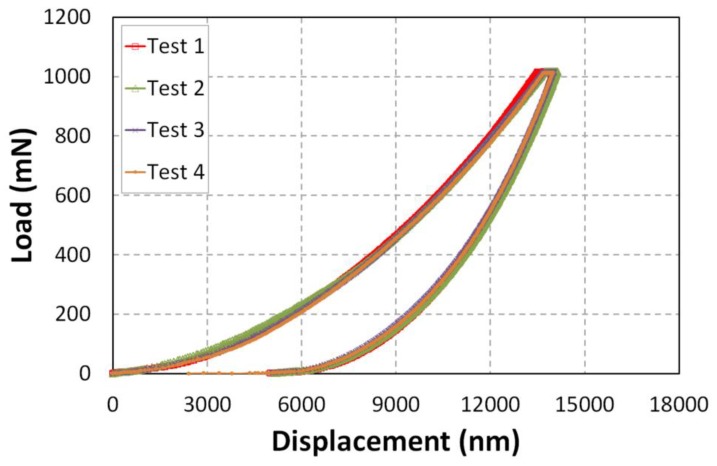
Indentation profile for epoxy matrix, 2.5 wt %NGP.

For a qualitative and quantitative study, it is necessary to ensure test reproducibility. Figure 10 shows the indentation profile for epoxy matrix with 2.5 wt % of graphene additives. Only a negligible difference was found between various indentation curves. From these curves, it appears that specimens show little difference in maximum indentation depths under the same load level, for example 13.96, 14.13, 14.02, and 13.99 µm. This can be explained by the indented zone, the presence of graphene particles in Epoxy matrix, graphene/Matrix interface and possibility to have a minor amount of pores in specimens.

### 4.4. Micro-Indentation Profiles

**Figure 11:**
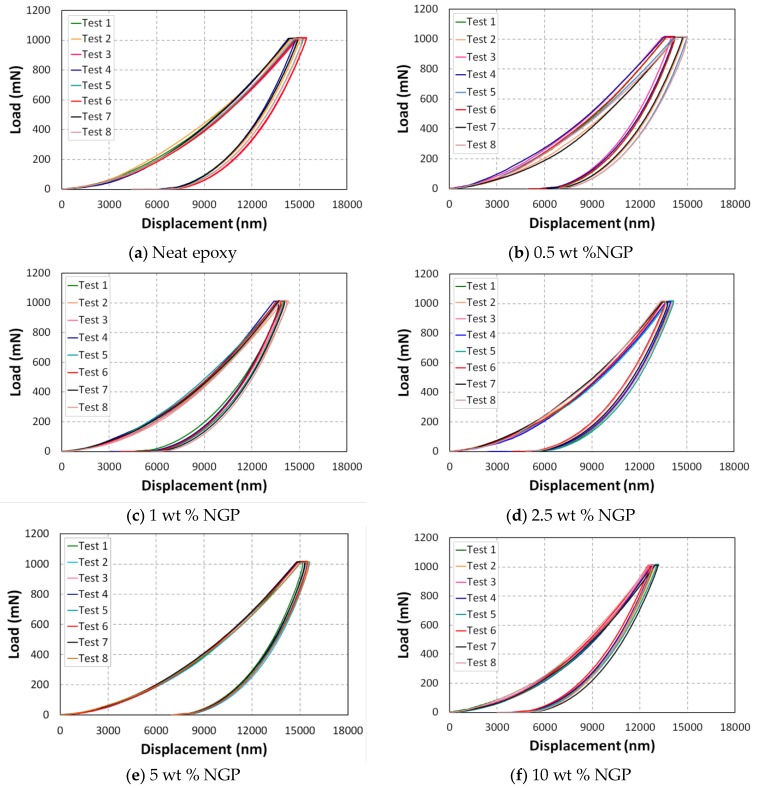
Indentation profile for epoxy matrix with graphene additives.

**Figure 12:**
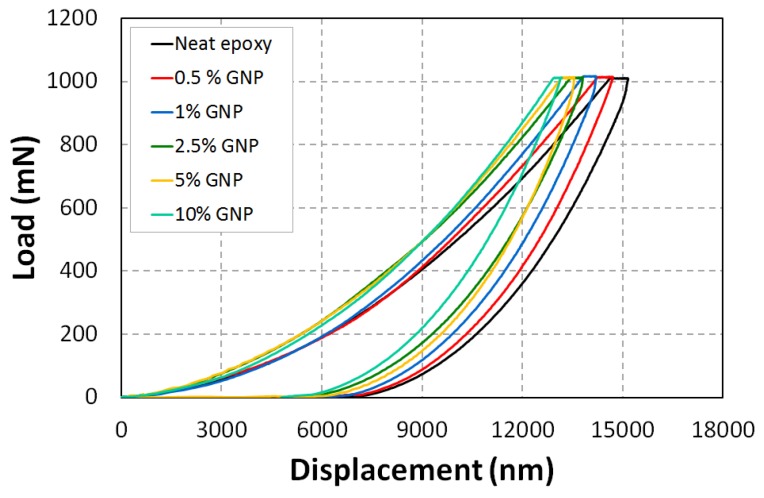
Graphene effect on local mechanical behavior, indentation tests.

The load–displacement curves for each graphene-based nanocomposite are shown in Figure 11. These load–displacement curves help to analyze the elastic to plastic behavior of the specimens at the surface. Figure 11 shows the P–δ relationships of NGP/epoxy samples measured using indentation apparatus. Compared to neat epoxy, the NGP/epoxy sample displays a significantly higher indentation load (P), e.g., the value of P is roughly increased by 3.7%, 8.2%, 19.6%, 21.8% and 22.7% for 0.5, 1, 2.5, 5, and 10 wt %respectively at δ = 10 μm, which means that the graphene produced a significant contribution to the overall indentation response of NGP/epoxy (Figure 12). The same observation can be made for the displacement of the indenter. Indeed, with the addition of graphene particles, there is an increase in the stiffness, and, subsequently, a decrease in displacement for a force of 1000 mN. In comparison to neat epoxy, the displacement decreases for 0.5, 1, 2.5, 5, and 10 wt % NGPs by 2.8%, 5.8%, 8.3%, 10.1% and 11.7%, respectively (Figure 12). For both Epoxy and Epoxy/NGP samples, the indentation deformation could not be recovered after unloading, which means that their deformations were not elastic; there is a difference between the loading and unloading curves. In this figure, it can be observed that as the concentration of the additives increased, the rigidity of the material increased.

**Figure 13:**
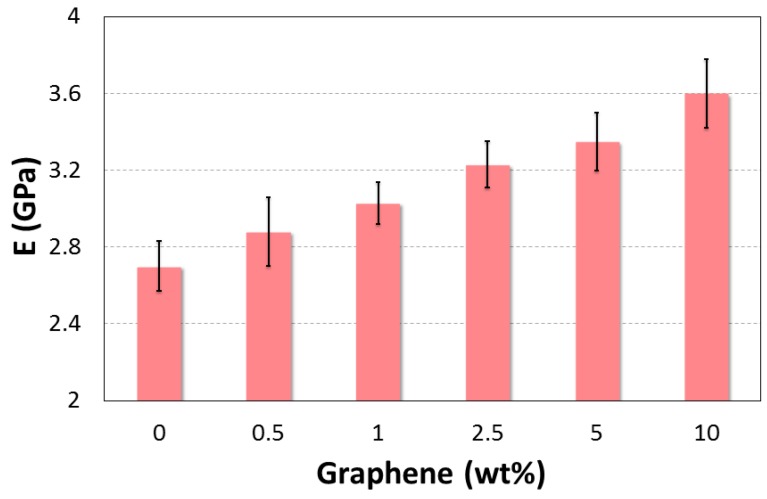
Effect of graphene additives on young modulus.

**Figure 14:**
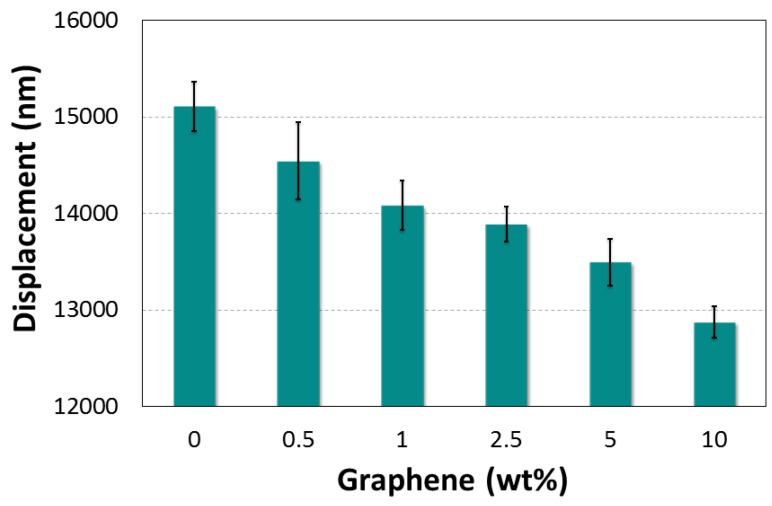
Effect of graphene additives on maximum displacement.

Figure 13 and Figure 14 combine the calculated Young’s modulus and the maximum displacement as a function of graphene weight fractions. The addition of graphene into epoxy matrix appears to have enhanced the interfacial resistance, thereby increasing the elastic modulus from 2.7 GPa to 3.6 GPa. This improvement is due to the high aspect ratio and intrinsic mechanical performance of graphene in comparison to the epoxy matrix.

As shown in Figure 14, the NGP/epoxy samples showed much less indentation depth than Neat Epoxy at 1000 mN. The lower indentation depth for the NGP/epoxy was affected by the percentage of NGP. The graphene additives, therefore, can significantly improve the indentation resistance of the polymer nanocomposite. The improved indentation resistance by NGP/epoxy can be identified in the loading phase and the hold periods at *P*_max_.

**Figure 15:**
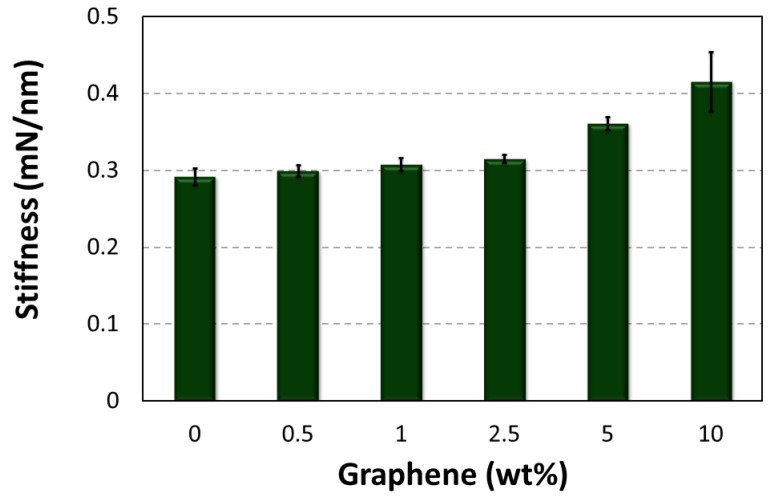
Effect of graphene additives on stiffness.

Figure 15 shows that the graphene-reinforced epoxy matrix have a much greater resistance to indentation than that of the individual stiffness of neat epoxy.

**Figure 16:**
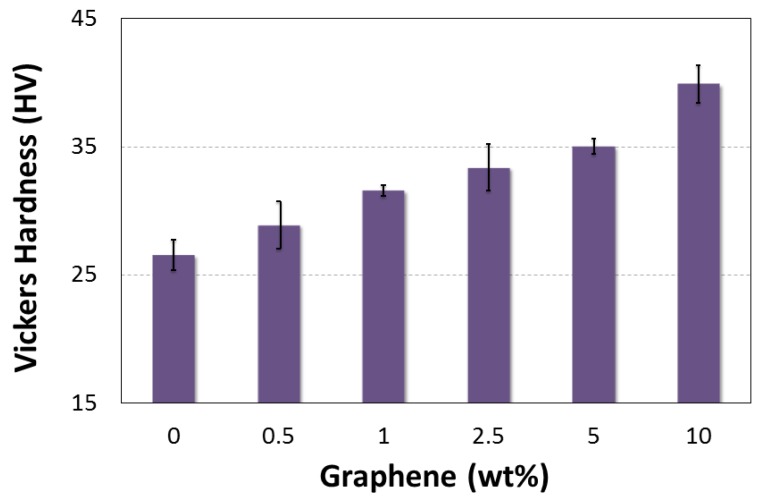
Effect of graphene additives on NGP/epoxy hardness.

Figure 16 show the effect of graphene content on the hardness of the NGP/epoxy nanocomposites. A significant improvement in hardness was observed with the addition of NGP. The neat epoxy samples exhibit a hardness of 26.56 kgf/mm^2^, which increases to 28.90 (8.83%), 31.59 (19%), 33.37 (26%), 35.02 (32%) and 39.89 (50%) with the addition of 0.5 1, 2.5, 5 and 10 wt % NGP, respectively. This increment in hardness is due to a good dispersion and interfacial bonding between NGP and the epoxy matrix.

## 5. Discussion

The load–displacement curves for the neat epoxy resin and various NGP/epoxy-based nanocomposites were analyzed. These curves were obtained by micro-indentation testing under a maximal load of 1000 mN. As a result, adding the graphene nanoparticles (NGP) to neat epoxy, the mechanical properties were altered. Indeed, the elastic modulus and the stiffness (slope for the unloading) were improved. The elastic modulus of the epoxy increased from approximately 2.7 GPa to 3.6 GPa upon the addition of 10 wt % of NGP to the epoxy resin. Our results also showed that the stiffness and hardness of NGP/epoxy samples increased by 42% and 40%, respectively. The values of mechanical properties (*E*, δ, *S* and *H*) are listed in Table 3. The reported results for each specimen represent an average of six or more different indentation tests. The hardness is defined as the resistance of the material surface to the plastic deformation that is induced by a normal load, and the elastic modulus is the material’s ability to recover its former shape. It was observed that adding NGP to the epoxy matrix causes the indenter to interact with more nanofillers.

## 6. Finite Elements Analysis

In the previous sections, the experimental results of the indentation tests are summarized. In this section, the numerical investigations performed using finite element methods are presented.

### 6.1. Generation and Meshing of Micro-Indentation Model

**Figure 17:**
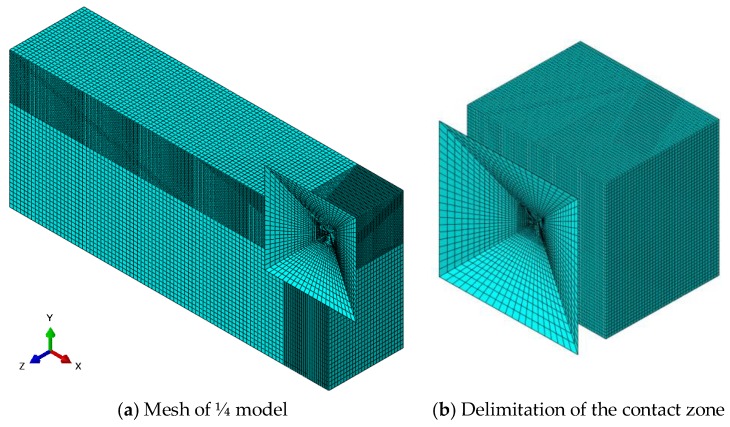
3D Finite element model for indentation test simulation.

A 3D structural solid element type C3D8R for specimen of composites based NGPs and epoxy matrix for modeling Vickers indenter was adopted by using Finite Element software ABAQUS. The mechanical properties are isotropic for epoxy matrix, NGPs and for the indenter (rigid) (Table 2). A homogenization procedure was necessary to evaluate the properties of the specimens with the different percentages of NGPs. The indentation was simulated as a contact between rigid indenter and elastic specimens; no elasto-plastic effect was considered. The specimen was fixed on the bottom and lateral sections, and the rigid indenter was animated by a translation movement in the direction of thickness. Figure 17a shows the mesh of ¼ models. As the effect of indentation is very localized, Figure 17b shows the studied part. We can also note the mesh used which is the result of the study of the mesh convergence. The mesh refinement around contact area was performed and, by considering a good estimation of the indentation contact area, the mesh size was optimized for rapid convergence. The mesh size was arranged to be more refined in the indentation site, i.e., under Vickers indenter, and coarse at the others zones.

The total mesh of indentation geometry was composed of 45,310 linear hexahedral elements of C3D8R for specimen, 4986 linear quadrilateral elements of R3D4 and 2 linear triangular elements of R3D3 for rigid indenter. Figure 17 shows the schematic representation of the finite element mesh of ¼ model and an enlarged view of the contact region.

### 6.2. First Validation of Numerical Models

**Figure 18:**
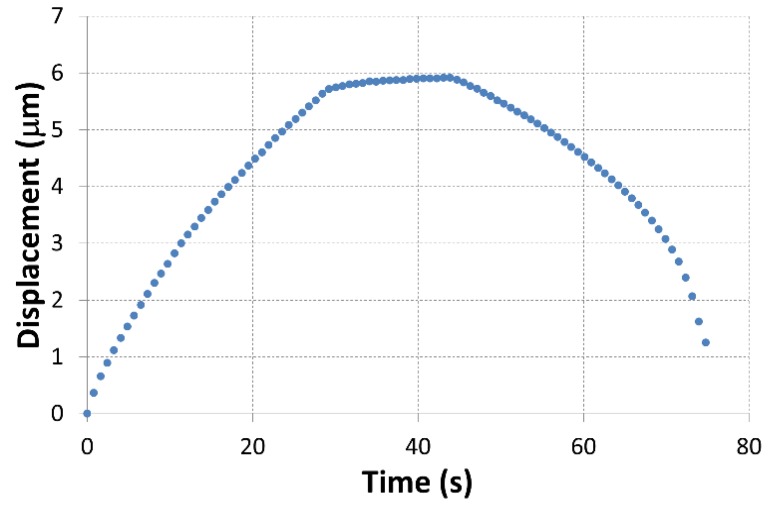
Displacement–time boundary condition applied to the numerical model.

**Figure 19:**
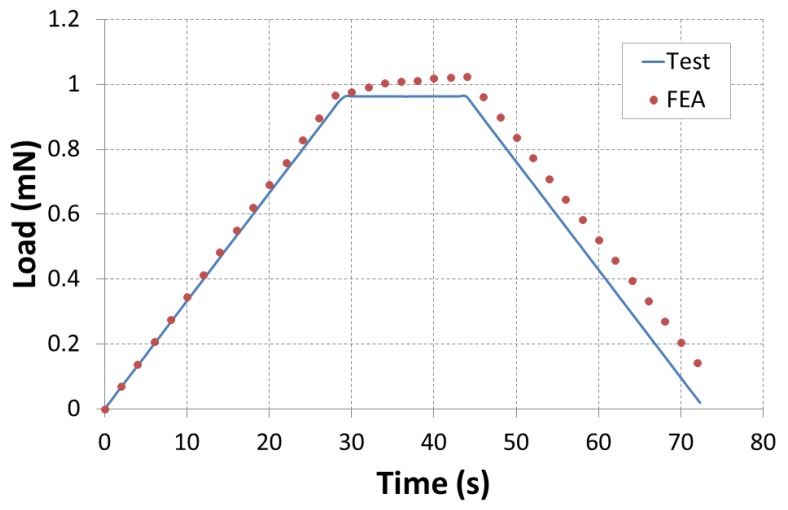
Correlation between the experimental and numerical load.

We developed a numerical model of micro-indentation of the composites based on the implicit scheme using Abaqus FEA software. The force at each increment time was examined and correlated to the experimental variations to validate our numerical model and enhance the convergence of results. The boundary condition applied to the indenter is the displacement resulting from the experimental results (Figure 18). The correlations between numerical and experimental load are presented in Figure 19. A good correlation was found between the two methods. Comparable to experimental variation, the numerical response presents three different zones: load phase, 15 s of pause and unloading phase. A small difference is noted in the unloading phase beyond 30 s of loading. This difference is due to the presence of damage. Thus, the numerical model could be employed to simulate the indentation test. No damage criteria were executed in the numerical model.

### 6.3. Modeling the Indentation Test

**Figure 20:**
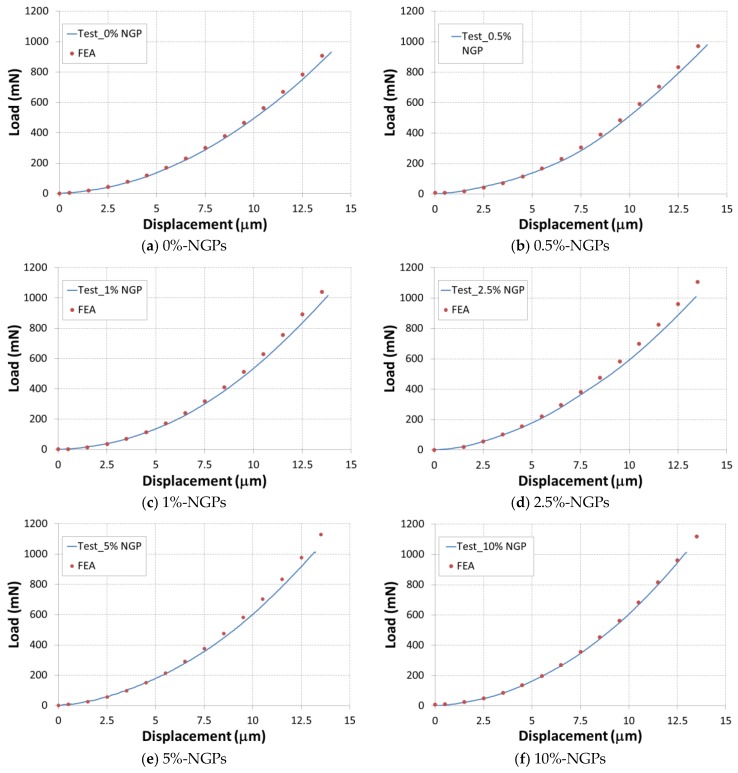
Experimental test and numerical simulations of micro-Indentation, loading zone.

Figure 20 presents the comparison between results of simulation and experiment for load–displacement curves. The profile of numerical model follows the experimental variations in elastic region. The maximum displacements of numerical and experimental variations are almost the same. We observed a small difference between the two curves. This is error due to the difference between the value of Young’s modulus and those determined from the micro-indentation tests. Finally, the numerical approach is suitable for simulating the effect of graphene nano-additives on the local mechanical behavior of derived polymer nanocomposites with Micro-indentation test.

## 7. Conclusions

Local mechanical properties of an epoxy matrix reinforced with graphene nanoparticles (NGP) were investigated using depth-sensing micro-indentation. The addition of low percentage weight of NGP was considered as part of a strategy to enhance the mechanical properties of the nanocomposites. The graphene additives were uniformly distributed into the epoxy matrix, forming a nanocomposite. The study focused on the effect of the concentration of NGPs on the mechanical performance. It was found that the addition of NGP to epoxy matrix reduces the indentation depth (δ) and increases the Young modulus (*E*), Stiffness (*S*) and hardness (*H*). Micro-indentation tools have provided a new method to evaluate the degree of dispersion of nano-additives and the local mechanical properties at the micrometric scale. The local mechanical properties can be considered representative of those of the bulk. This study shows that the indentation test could be an excellent technique in characterizing the mechanical properties of composites laminate reinforced with graphene nano-additives. It seems that the local mechanical properties of nanocomposite samples were improved as the amount of nano-additives increased. Based on the curve displacement and surface imaging, the influence of nano-additives on the overall plastic mechanical behavior is noticeable. A proper numerical model was developed for validating the experimental results. A small difference between two approaches was remarked and defined by the projected contact area and the graphene nano-additive distribution. It seems that graphene tends to improve the fracture toughness of composite and interfacial resistance.

## Figures and Tables

**Table 1:** Feed roller speed.

Formula	Nanofiller (%)	Feed Roll Speed (rpm)
1	0	333
2	0.5 and 1	400
3	2.5	480
4	5	550
5	10	600

**Table 2:** Mechanical properties of the constituents.

Materials	*E* (GPa)	υ
**Epoxy matrix**	2.72	0.3
**Graphene**	1030	0.19

**Table 3:** Mechanical properties versus graphene weight fractions.

Graphene Additives NGP (wt %)							
NGP (wt %)		0	0.5	1	2.5	5	10
**E (GPa)**	Mean	2.7	2.88	3.03	3.25	3.35	3.6
	St. Dev	0.13	0.18	0.11	0.12	0.14	0.18
	Rise (%)	-	+6.67	+12.22	+19.63	+24.07	+33.33
**δ max (μm)**	Mean	15.105	14.54	14.08	13.88	13.49	12.87
	St. Dev	0.25	0.40	0.25	0.18	0.24	0.16
	Fail (%)	-	−3.72	−6.76	−8.06	−10.66	−14.75
**S (mN/µm)**	Mean	292.2	300	308	315.4	361.2	415.8
	St. Dev	10.94	7.07	8.36	5.55	8.35	38.83
	Rise (%)	-	+2.67	+5.41	+7.94	+23.61	+42.30
**H (kgf/mm^2^)**	Mean	26.56	28.91	31.59	33.38	35.02	39.89
	St. Dev	1.20	1.84	0.40	1.80	0.58	1.44
	Rise (%)	-	+8.84	+18.94	+25.67	+31.86	+50.21
